# Advances in virus-induced flowering in tomato

**DOI:** 10.1093/jxb/erad407

**Published:** 2023-12-20

**Authors:** Francesca Bellinazzo

**Affiliations:** Laboratory of Molecular Biology, Wageningen University and Research, 6708 PB, Wageningen, The Netherlands; Bioscience, Wageningen Plant Research, Wageningen University and Research, 6708 PB, Wageningen, The Netherlands

**Keywords:** Florigen, Flowering Locus T, lifecycle shortening, potato virus X, *Solanum lycopersicum*, speed breeding, tomato, virus-induced flowering

## Abstract

This article comments on:

**Deng Y, Yarur-Thys A, Baulcombe DC.** 2024. Virus-induced overexpression of heterologous *FLOWERING LOCUS T* for efficient speed breeding in tomato. Journal of Experimental Botany **75**, 36–44.

**Many plants use information on day length, which is sensed in the leaves, to time their flowering response at the shoot apical meristem (SAM). What makes this mechanism possible is a transmissible molecule called florigen, a small protein encoded by the gene *FLOWERING LOCUS T* (*FT*). Based on this knowledge, virus-induced flowering (VIF) can be applied to promote the change to the reproductive phase and to facilitate the breeding of plants with long juvenility or unsynchronized flowering time. In their study, Deng *et al*. (2024) describe a potato virus X (PVX)-based method to transiently deliver the florigen in tomato plants and thereby control their flowering time.**

Viral vectors have been widely used for transient genetic manipulation to study gene function in plants. This approach is especially useful as an alternative to the generation of stable mutant lines in recalcitrant species. The most common virus-based technology is the so-called virus-induced gene silencing (VIGS), which turns the immune response of the plant host against its own transcripts (Lu *et al*., 2003). In essence, a viral vector is engineered with a fragment of the target gene. Upon infection, the virus produces the engineered transcript in double-stranded form. The presence of double-stranded RNAs triggers the plant’s post-transcriptional gene-silencing machinery, which is sequence specific. Consequently, targeted RNA degradation occurs, resulting in down-regulation of the endogenous target gene together with the engineered fragment produced by the virus (Rössner *et al*., 2022). The use of viral vectors not only allows gene down-regulation; its opposite, overexpression, can also be obtained. In fact, by inserting the full-length coding region of the gene of interest into a viral vector, transient ectopic expression can be obtained. This technology is called virus-induced overexpression (VOX) (Rössner *et al*., 2022).

Building upon these research methods, a new breeding tool has been developed to induce flowering on demand and to facilitate crossing. A tailored version of the VOX technology, VIF (McGarry *et al*., 2017), uses FT to harness its florigenic power (Fig. 1). The key aspect behind the success of this method can be found in the mobile nature of FT. In fact, a common bottleneck of virus-based systems is the inability of a viral infection to reach the correct plant tissue. This problem is especially relevant for meristematic areas including the SAM, from which most viruses are effectively excluded (Bradamante *et al*., 2021). Although the meristematic barrier against viruses is crucial for blocking vertical transmission of the infection to the next generation (and therefore allowing transient manipulation to remain as such), it can, in principle, prevent the manipulation of meristematic genes. By using a systemically transmissible inducer of flowering, the VIF technique can exert its effect in virus-free meristematic cells.

**Fig. 1.**
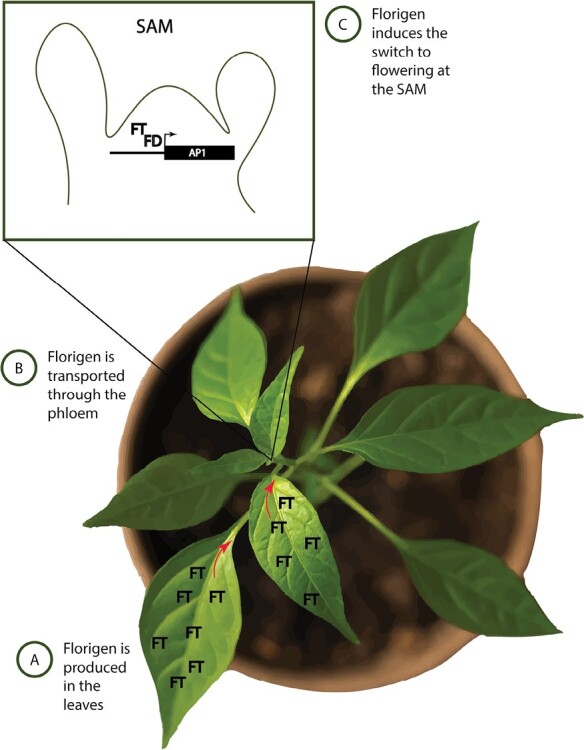
Illustration showing the mode of action of florigen. (A) Florigen, the gene *FT*, is expressed and translated in leaf vasculature, specifically in phloem companion cells. In many plant species, *FT* expression occurs in response to photoperiod. (B) The FT protein is transported systemically through the plant phloem (indicated by the red arrows). (C) On reaching the shoot apical meristem (SAM), FT binds to the bZIP transcription factor FD in a higher-order complex, allowing the expression of flower meristem identity genes (exemplified by AP1 in the figure), which mediate the change to the reproductive phase (Turck *et al*., 2008; Taoka *et al*., 2011; Collani *et al*., 2019).

VIF has been applied to multiple crops, including melon (*Cucurbita moschata*), cotton (*Gossypium hirsutum*), soybean (*Glycine max*), and grapevine (*Vitis vinifera)*, demonstrating that FT can be delivered and flowering can be successfully induced in these species (McGarry *et al*., 2017; Maeda *et al*., 2020). Notably, VIF is effective even during the juvenile stage, as it has been demonstrated in apple (*Malus domestica*) (Yamagishi *et al*., 2011) and in orange (*Citrus sinesis*) (McGarry *et al*., 2017). Nevertheless, there is no single solution for performing VIF in all plant species: in fact, whereas FT is highly conserved in angiosperms (Bennett and Dixon, 2021; Liu *et al*., 2023), most plant viruses have a relatively narrow host range (McLeish *et al*., 2019). For this reason, VIF requires customization, which can be challenging. In their study, Deng *et al*. (2024) successfully optimized the VIF method for tomato (*Solanum lycopersicum*). Tomato is a major vegetable crop, accounting for 16% of the total global vegetable production in 2020 (FAOSTAT, 2022). The viral vector used in their study is based on PVX, a monopartite virus, which is advantageous because only one viral molecule needs to be transformed into each plant cell for the system to function.

To prove that the PVX vector can effectively be delivered in tomato plants, a VIGS system was first designed to silence the gene *PHYTOENE DESATURASE* (*PDS*) in tomato. *PDS* is commonly used as a visual marker because its silencing causes photobleaching, and therefore the sites of viral infection and spread can be easily identified by the whitening of the infected tissues (Romero *et al*., 2011; Killiny, 2022). In parallel, the presence of the PVX coat protein and of the 25K protein, which is essential for movement of the virus inside the plant (Leshchiner *et al*., 2008), was verified at the RNA level by quantitative PCR. Subsequently, two versions of PVX-based VIF vectors were engineered, one carrying the Arabidopsis *FT* coding region and the other with the coding sequence of the main tomato florigen *SINGLE FLOWER TRUSS* (*SFT*). Treated tomato plants flowered 28–34 days after inoculation, approximately 10 days earlier than untreated controls. Moreover, VIF-treated plants produced >85% more flowers per truss and, ultimately, more ripe fruits (>62% increase). These results are in line with what is observed in *sft* mutants, where a late-flowering phenotype is paired with single-flower trusses. In striking contrast with the Arabidopsis *ft* mutants (Périlleux *et al*., 2019), the single-flowered *sft* phenotype is due to the fact that SFT not only promotes flower induction but is also necessary to maintain the reproductive identity of meristems, which otherwise would reverse to the vegetative state (Molinero-Rosales *et al*., 2004).

Interestingly, enhanced VIF-related effects were observed when using Arabidopsis FT instead of the native SFT. Although this might seem surprising, the authors explain the milder phenotypes by showing that the presence of native *SFT* transcripts partially triggers silencing as well as overexpression. The use of heterologous genes to avoid silencing of the corresponding endogenous gene might be more generally useful when VOX is used as a research tool. Finally, PVX-infected plants produced healthy seeds that germinated into virus-free plants. This excludes the possibility of vertical transmission of the viral vector, indicating that this system is, in principle, safe to use for breeding.

In conclusion, a reliable method has been established to transiently induce flowering in tomato. This advancement has been made possible thanks to the discovery of florigen (Chailakhyan, 1936), obtained through fundamental research conducted around 100 years ago, sometimes in difficult circumstances (Romanov, 2012). This shows that the effects of a past great discovery keep reverberating in a cascade of new conceptualization and technology, ultimately sustaining the production of new plant varieties that have potential to withstand the climatic crisis today and in the future.
